# Global spread and evolutionary trajectories of HIV-1 CRF01_AE: Insights from phylogenetic analysis

**DOI:** 10.1371/journal.pone.0360404

**Published:** 2026-10-08

**Authors:** Xingguang Li, David Jorgensen, Haizhou Liu, Qing Nie, Callum Bates, Quan Gu

**Affiliations:** 1 Ningbo No 2 Hospital, Ningbo, China; 2 Guoke Ningbo Life Science and Health Industry Research Institute, Ningbo, China; 3 MRC Centre for Global Infectious Disease Analysis, Department of Infectious Disease Epidemiology, School of Public Health, Imperial College London, London, United Kingdom; 4 State Key Laboratory of Virology and Biosafety, Wuhan Institute of Virology, Chinese Academy of Sciences, Wuhan, China; 5 Microbiology Laboratory, Weifang Center for Disease Control and Prevention, Weifang, China; 6 MRC-University of Glasgow Centre for Virus Research, Glasgow, United Kingdom; Nazarbayev University School of Medicine, KAZAKHSTAN

## Abstract

HIV-1 circulating recombinant form (CRF) 01_AE has driven major HIV epidemics across Southeast and East Asia for decades, yet the mechanisms underlying its long-term regional spread remain poorly resolved. Using near-complete genome sequences and Bayesian phylogeographic reconstruction, we traced the evolutionary history of CRF01_AE from its origin in Central Africa to its expansion across Asia. Our results uncover a decades-long transmission corridor linking Africa, Thailand, and China, in which CRF01_AE was introduced into Thailand in the early 1980s and subsequently disseminated across Southeast Asia and into China through repeated cross-border movements. Many viral lineages circulated for years before detection, revealing substantial cryptic transmission prior to surveillance. Once established, transmission clusters persisted for decades, sustaining local epidemics long after their initial introduction. These findings highlight the public health importance of early molecular surveillance and coordinated regional strategies to detect emerging lineages and interrupt cross-border transmission.

## Introduction

HIV-1 CRF01_AE is one of the most widely disseminated circulating recombinant forms (CRFs) of HIV-1 and plays a critical role in the global HIV pandemic, particularly in Southeast and East Asia [1,2]. HIV-1 CRF01_AE was the first CRF to be identified and is believed to have emerged from a recombination event between subtype A (likely from Central Africa) and subtype E, a designation once used to describe a viral strain not found as a pure subtype in humans [3–5]. HIV-1 CRF01_AE originated in Central Africa and was detected in samples from the Democratic Republic of Congo dating back to the mid-1980s [6]. However, the first documented strains were first identified in 1989 in northern Thailand, among female commercial sex workers [7–9]. The strain subsequently spread across multiple risk groups within Thailand and neighboring countries [10–14]. HIV-1 CRF01_AE is a key constituent of at least 61 recognized CRFs globally (https://www.hiv.lanl.gov/components/sequence/HIV/crfdb/crfs.comp).

In recent decades, HIV-1 CRF01_AE has contributed significantly to various transmission networks including heterosexual contact, men who have sex with men (MSM), and people who inject drugs (PWID). Its unique evolutionary history, distinct biological properties, and wide geographic distribution underscore the importance of CRF01_AE in understanding HIV-1 transmission dynamics, pathogenesis, and control strategies. Despite extensive epidemiological research [12,13,15–19], the spatial-temporal dynamics and evolutionary history underlying the transnational spread of CRF01_AE remain poorly understood.

In this study, we employed a Bayesian phylogeographic framework integrating near-complete genome sequences to reconstruct the origin, migration routes, and transmission dynamics of CRF01_AE. Our analysis traces the earliest diversification of the virus to Central Africa, followed by its rapid expansion in Thailand during the 1980s and 1990s, where it became the dominant strain driving the early heterosexual and commercial sex epidemics, and was subsequently introduced into neighboring countries including Vietnam and China.

Despite ongoing prevention efforts, CRF01_AE continues to evolve, posing persistent public health challenges and necessitating continued surveillance and molecular epidemiological research. By characterizing the geographic structure and estimating the timing of key migration events, our findings provide critical insights into the factors shaping regional CRF01_AE epidemics and highlight the enduring influence of historical transmission routes on the current genetic diversity of CRF01_AE.

## Materials and methods

### Genome sequence curation of HIV-1 CRF01_AE

A total of 334 near full-length genome (NFLG) sequences of HIV-1 CRF01_AE, each representing a unique individual and annotated with sampling date and geographic origin, were curated from the Los Alamos National Laboratory (LANL) HIV Sequence Database (http://www.hiv.lanl.gov), as of July, 2013, as previously described [1]. The sequences, collected between 1990 and 2011, encompass nine geographic regions: China (*n* = 154), Thailand (*n* = 134), Vietnam (*n* = 33), the United States (*n* = 5), the Central African Republic (*n* = 3), Japan (*n* = 2), Afghanistan (*n* = 1), Hong Kong (*n* = 1), and Indonesia (*n* = 1). The geographic distribution of HIV-1 CRF01_AE was visualized and annotated using Microreact [20] (S1 Fig).

### Phylogenetic analyses

A Maximum-likelihood phylogenetic tree was inferred using PhyML v3.3.20180621 [21] under the GTR + Γ4 nucleotide substitution model. Node support was evaluated through 1000 bootstrap replicates. Phylogenetic clusters were delineated using Cluster Picker v1.2.5 [22], based on the following criteria: Initial Threshold of 900, Main Support Threshold of 900, Genetic Distance Threshold of 6.67%, and Cluster Size Threshold of 3. Genetic distances between and within phylogenetic clusters were calculated in MEGA v12.0.11 [23,24] using the Maximum Composite Likelihood model [25] with 1000 bootstrap replicates. The rate variation among sites was modelled with a gamma distribution (shape parameter = 4) [26]. Genetic distances within and between phylogenetic clusters were visualized using a heatmap generated with the *pheatmap* package (v1.0.13) in R (v4.5.3) and RStudio (v2026.01.1 + 403).

To evaluate the presence of a temporal signal, we assessed the correlation between root-to-tip genetic distances and sampling years using TempEst v1.5.3 [27]. The input tree was the maximum-likelihood tree generated using PhyML v3.3.20180621 [21] as mentioned above. We subsequently employed a Bayesian phylogenetic framework to jointly estimate the time to the most recent common ancestor (tMRCA), evolutionary rate, and demographic history of CRF01_AE. Analyses were conducted using an uncorrelated lognormal (UCLN) [28] relaxed molecular clock model, the GTR + Γ4 nucleotide substitution model, and a Bayesian Skygrid [29] coalescent prior, as implemented in BEAST v1.10.4 [30]. The Markov chain Monte Carlo (MCMC) run was performed for 1 billion iterations and logged parameters every 100,000 iterations. MCMC convergence was checked in Tracer v1.7.2 [31], and the effective sample sizes for continuous parameter were ≥200.

### Phylogeographic analyses

Using the posterior distribution of trees from the above phylogenetic analyses as an empirical tree set, we performed an asymmetric continuous-time Markov chain (CTMC) discrete trait analysis (DTA) [32] in BEAST v1.10.4 [30]. We implemented Bayesian stochastic search variable selection (BSSVS) to identify statistically supported non-zero transition rates [32]. Well-supported transition rates were identified by Bayes factors (BF) with a cut-off of 3 using SpreaD3 v0.9.7.1 [33]. We adopted the BF interpretation scale from a previous study [34], where BF values between 3 and 20 indicates positive evidence, values between 20 and 150 indicate strong evidence, and values greater than 150 indicate very strong evidence. We employed Markov jump analysis to quantify the number of transition events between discrete trait states across the posterior set of trees using a robust counting approach [35–37]. We also employed Markov reward analysis to estimate the expected waiting time spent in each discrete trait state along the phylogeny. The Markov chain Monte Carlo (MCMC) run was performed for 100 million iterations and logged parameters every 10,000 iterations. MCMC convergence was assessed in Tracer v1.7.2 [31], ensuring that effective sample sizes for all continuous parameters were ≥200. Maximum clade credibility (MCC) trees were summarized using TreeAnnotator v1.10.4 after discarding the initial 10% as burn-in. To obtain posterior summaries of Markov jump counts and their associated timings, we employed the Tree Markov Jump History Analyzer tool in BEAST v1.10.4 [30], applied to the posterior tree distribution annotated with Markov jumps [38]. The MCC phylogenetic tree was visualized and annotated using the R package ggtree v4.1.1.4 [39–41] in R (v4.5.3) and RStudio (v2026.01.1 + 403).

## Results

### The phylogeny of HIV-1 CRF01_AE strains

Phylogenetic analysis of HIV-1 CRF01_AE strains using a maximum likelihood approach revealed the presence of 10 distinct phylogenetic clusters (S1 Fig), as previously reported [1]. We further estimated the average evolutionary divergence within and between these clusters (S2 Fig). Among these, CRF01_10 AE exhibited the smallest average within-cluster divergence (0.3%), whereas CRF01_8 AE displayed the largest (6.4%). The smallest average inter-cluster divergence was observed between CRF01_2 AE and CRF01_9 AE (4.4%), while the largest divergence occurred between CRF01_4 AE and CRF01_8 AE (7.6%).

As shown in S3 Fig, the linear regression of root-to-tip genetic distances against sampling dates revealed relatively strong temporal signal with no clear outliers (i.e., clock-like evolution) for the 334 NFLG sequences of HIV-1 CRF01_AE (correlation coefficient = 0.91, *P* < 0.0001), consistent with previous findings [1].

Bayesian phylogenetic analysis revealed a median substitution rate of 4.7×10^−3^ substitutions per site per year (95% highest posterior density [HPD] interval: 4.4×10^−3^–4.9×10^−3^) (Fig 1). The median tMRCA was estimated to be October 6, 1973 (95% HPD interval: March 18, 1971–February 10, 1976), consistent with previous findings [1]. Linear regression analysis indicated a moderate positive correlation between evolutionary rate and tMRCA (correlation coefficient = 0.395, P < 0.0001).

**Fig 1:**
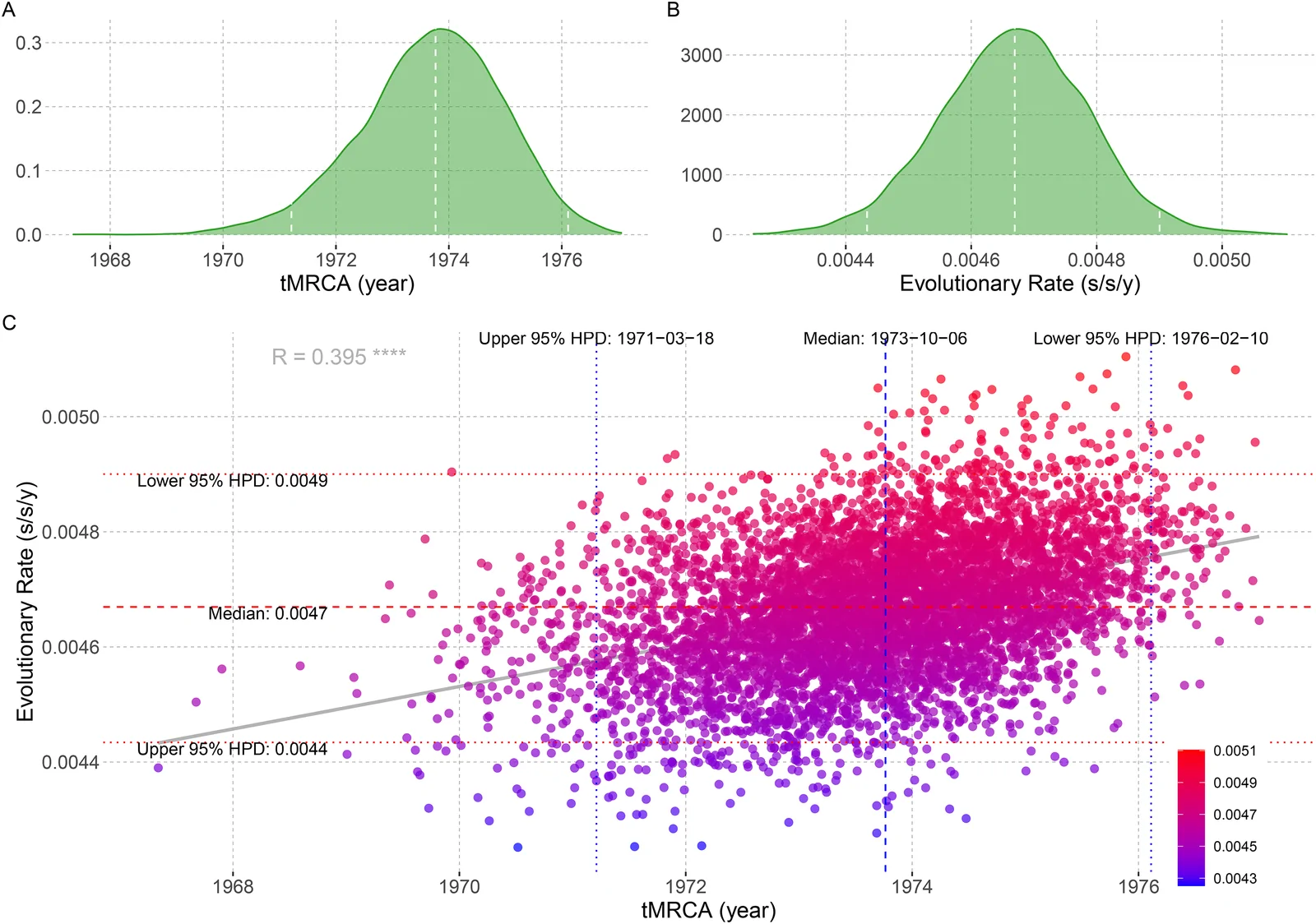
Evolutionary dynamics of HIV-1 CRF01_AE. (A) Density plot of tMRCA. (B) Density plot of evolutionary rates (substitutions per site per year). (C) Correlation between time to the most recent common ancestor (tMRCA) and evolutionary rate. The color bar represents evolutionary rates, with warmer colors corresponding to faster rates.

To reconstruct the historical population dynamics of HIV-1 CRF01_AE, we applied a Bayesian Skygrid [29] coalescent prior, which allows for flexible inference of temporal changes in effective population size. The effective population size exhibited a complex trajectory (S4 Fig), with two distinct phases of growth (1984–1992 and 1997–2006) separated by a period of decline (1992–1997).

### Discrete trait dispersal of HIV-1 CRF01_AE

To elucidate the spatiotemporal dynamics of HIV-1 CRF01_AE, we employed an asymmetric discrete trait diffusion model [32]. First, we investigated the geographic location origin of HIV-1 CRF01_AE. Inferred ancestral state reconstruction revealed the Central African Republic as the most probable (99.8%) geographic origin (posterior probability = 99.8%; Fig 2), consistent with prior findings [1]. The most probable geographic origins for CRF01_1AE to CRF01_10AE were inferred to be China, Vietnam, China, China, Thailand, China, China, China, Thailand, and Thailand, respectively (Figs 2 and 3A). The estimated tMRCAs ranged from 1987 to 1998, indicating that most clusters emerged during the late 1980s and 1990s. Phylogeographic reconstruction revealed multiple introductions, with most clusters originating from Thailand (6/10), one from Vietnam, and three likely arising within China (Fig 3A). Substantial delays were observed between viral introduction and subsequent cluster formation and detection. The estimated importation lag (time between introduction and cluster tMRCA) ranged from 0.75 to 6.1 years, indicating that viral lineages circulated for several years before establishing detectable transmission clusters. The detection lag (time between introduction and the first sampled sequence) ranged from approximately 7–21 years, suggesting prolonged periods of undetected transmission. Once established, clusters exhibited long-term persistence, with estimated persistence times ranging from approximately 8–21 years, consistent with sustained local transmission following the initial introductions (Fig 3A).

**Fig 2:**
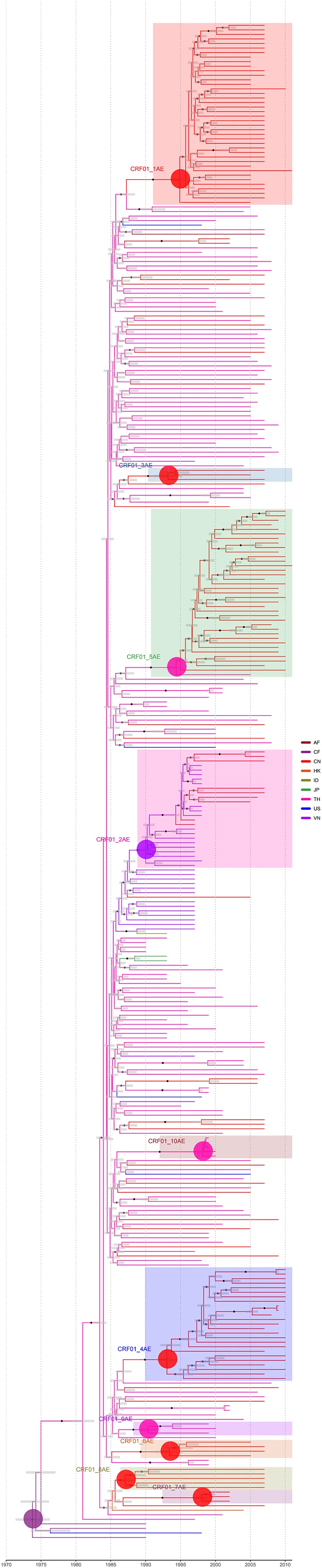
Maximum clade credibility (MCC) tree inferred from near-complete genome sequences of HIV-1 CRF01_AE. The inferred geographic origins of the ancestral HIV-1 CRF01_AE and phylogenetic clusters CRF01_1 AE to CRF01_10 AE are indicated by colored circles. Only internal nodes with posterior probability support ≥0.99 are indicated by black solid circles.

**Fig 3:**
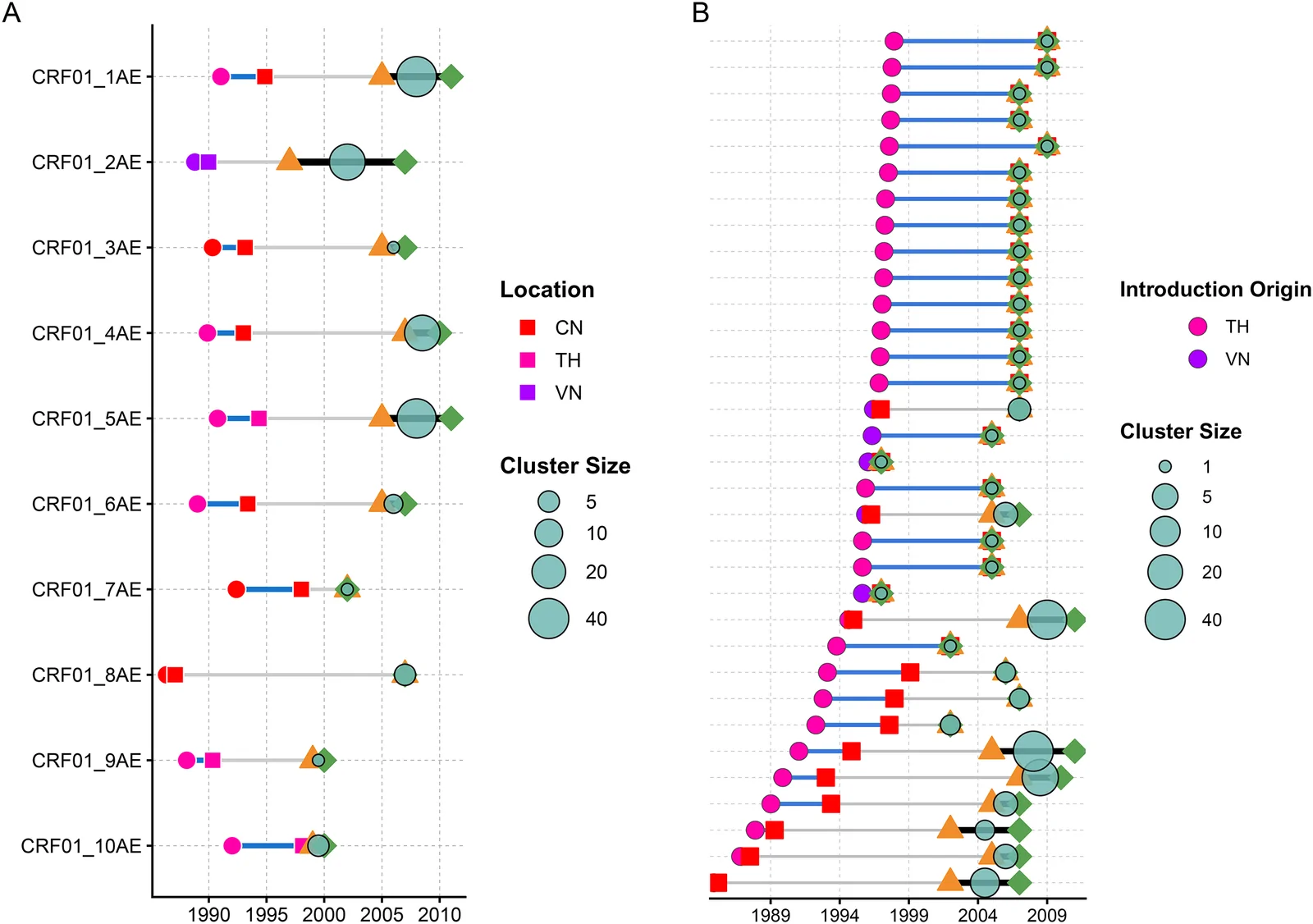
Timelines of HIV-1 CRF01_AE phylogenetic and transmission clusters. (A) Timeline of HIV-1 CRF01_AE phylogenetic clusters. Each row represents a cluster (CRF01_1 AE–CRF01_10 AE). Key events are indicated by symbols: circles, estimated introduction dates (colored by ancestral location: TH = Thailand, VN = Vietnam, CN = China); squares, cluster tMRCAs (colored by the most posterior probability of inferred location: TH = Thailand, VN = Vietnam, CN = China); orange triangles, first sampled sequence; green diamonds, last sampled sequence. Segment lines depict intervals: grey, tMRCA to first sampled sequence; blue, introduction to tMRCA; black, sampling period. Point size at cluster midpoint is proportional to cluster size. (B) Timeline of local HIV-1 CRF01_AE transmission clusters in China. Each row represents a distinct transmission cluster. Blue segments indicate the interval from introduction to tMRCA; grey segments, tMRCA to first sampled sequence; black segments, sampling period between the first and last sequences. Symbols denote key events: viral introduction (circles, colored by inferred origin: TH = Thailand, VN = Vietnam), cluster tMRCA (red squares), first sampled sequence (orange triangles), last sampled sequence (green diamonds), and cluster size (bubble size proportional to number of sequences).

Second, we estimated the total number of viral migration events between geographic locations (Fig 4A). The largest number of inferred migration events originated from Thailand to China, followed by migrations from Vietnam to China, Thailand to the United States, and Thailand to Vietnam.

**Fig 4:**
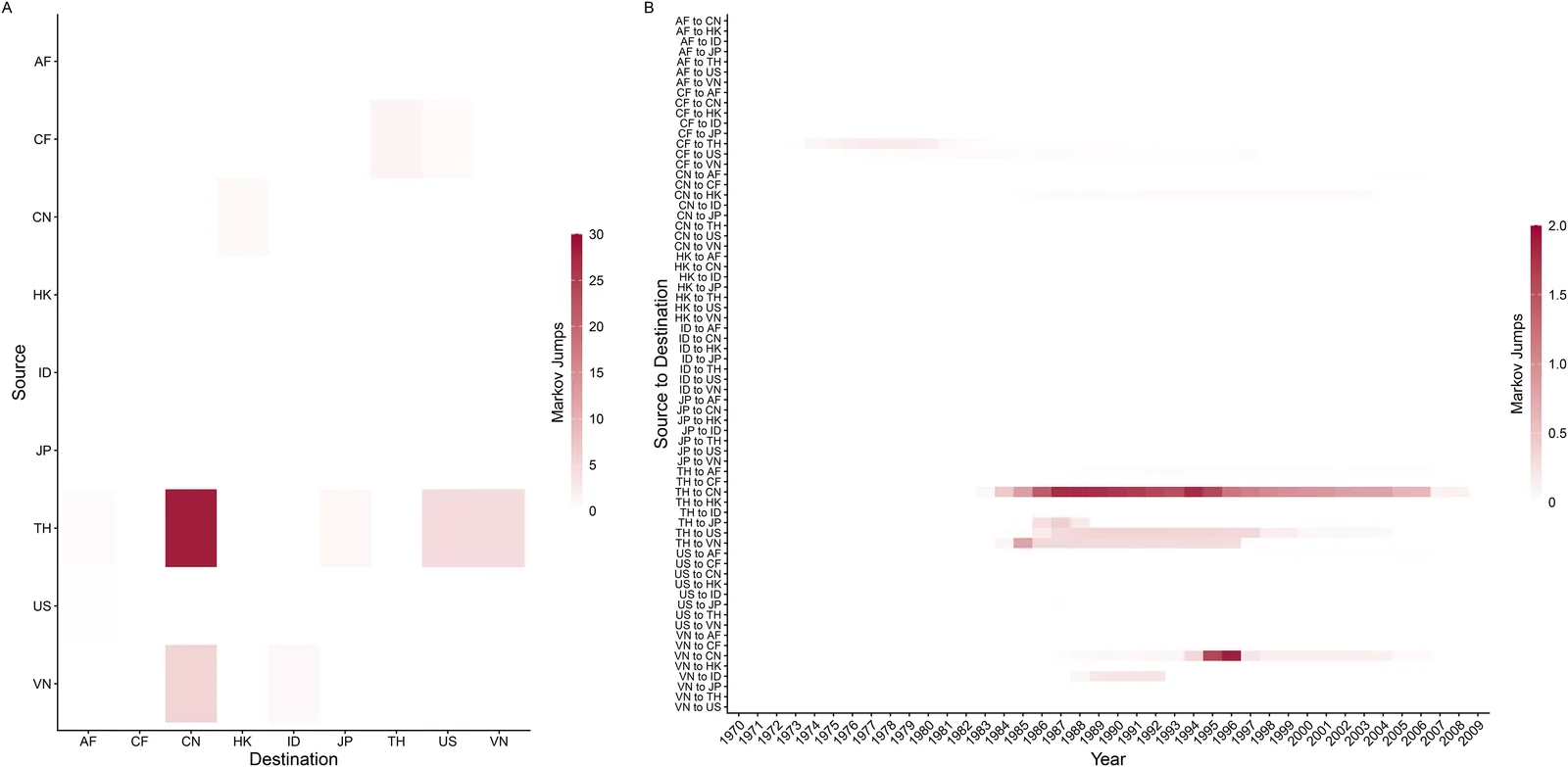
Geographic and temporal dynamics of HIV-1 CRF01_AE migration. (A) Estimated counts of geographic transition events inferred from ancestral state reconstruction, representing overall viral migration between regions. (B) Time-resolved estimates of geographic transitions inferred from ancestral state reconstruction, illustrating the changing patterns of viral movement between locations over time.

Third, we examined the temporal distribution of inferred viral migration events between geographic locations. The majority of migration events from Thailand to China were estimated to have occurred between 1986 and 1995 (Fig 4B). In contrast, migration events from Vietnam to China were primarily concentrated between 1995 and 1996, while those from Thailand to Vietnam predominantly occurred in 1985.

Fourth, we estimated the amount of time spent in each geographic location between transitions. In 1990, Thailand showed the highest mean duration, accounting for 73.2% of the trunk reward proportion, while China and Vietnam had lower proportions of 12.7% and 9.79%, respectively (S5 Fig). By 2000, the mean duration shifted predominantly to China, which contributed 54% of the trunk reward proportion, with Thailand and Vietnam contributing 44.6% and 4.19%, respectively. In 2010, China again exhibited the highest mean duration, accounting for 99.7% of the trunk reward proportion, while Thailand’s contribution decreased to 2.72%. Enumeration of the cumulative number of transitions between geographic locations over time revealed that China experienced more frequent and continuous jumps from Thailand than from Vietnam (S6 Fig).

Fifth, to illustrate the specific transmission route of HIV-1 CRF01_2AE from Vietnam to China, isolate 05GX001 was selected as a representative case. The Markov jump spatial trajectory plot of this isolate revealed an ancestral transition path originating in the Central African Republic, followed by transitions to Thailand, then Vietnam, and subsequently China (S7 Fig).

Sixth, we visualized the geographic dispersal of HIV-1 CRF01_AE using SpreaD3 v0.9.7.1 [33]. The spatial diffusion patterns (BF > 3, posterior probability > 0.79) identified Thailand as the epicenter following introduction from the Central African Republic, with subsequent spread to China, Vietnam, Japan, and the United States (Fig 5). Notably, HIV-1 CRF01_AE was introduced from Thailand to Vietnam, and subsequently to China.

**Fig 5:**
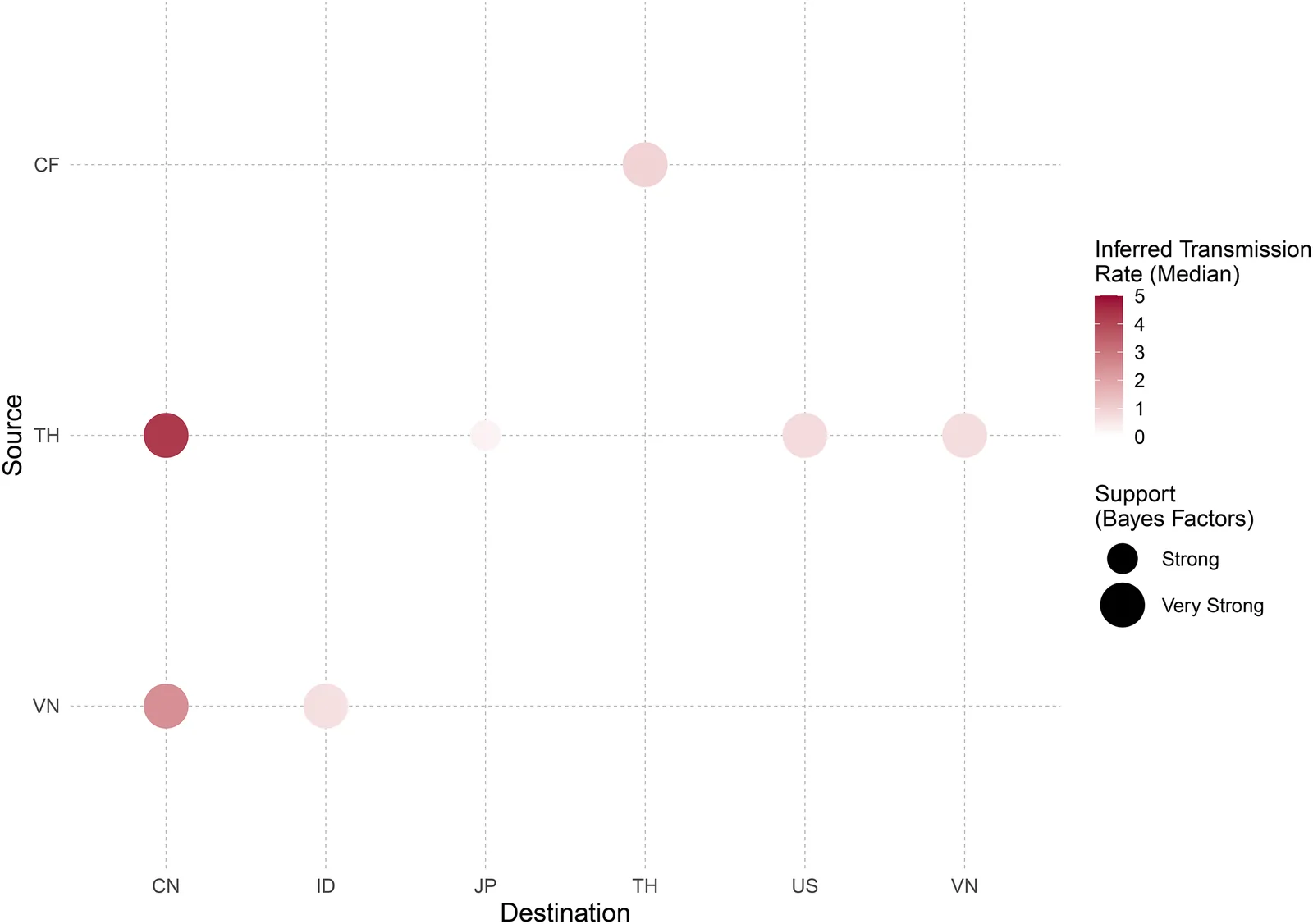
Global transmission rates of HIV-1 CRF01_AE inferred from a standard discrete phylogeographic analysis. The resulting transmission rate matrix is shown, highlighting only well-supported transitions identified by the standard discrete phylogeographic reconstruction (Bayes factor > 3 and posterior probability > 0.79).

Seventh, phylogeographic reconstruction identified 33 independent introductions into China, including 21 singleton lineages and 12 transmission clusters comprising 133 sequences. Cluster sizes ranged from 2 to 40 sequences, with three large clusters (>10 sequences) containing 25, 36, and 40 sequences, respectively (Fig 3B). Phylogeographic inference indicated that most introductions originated from Thailand (28/33), while five were inferred to originate from Vietnam. The estimated introduction times ranged from 1984 to 1997, and cluster tMRCAs ranged from 1985 to 2008, indicating that most local transmission lineages were established from the late 1980s to the 2000s. Substantial delays were observed between viral introduction and detection. The estimated importation lag ranged from 0.31–11.4 years. The detection lag ranged from 0.95–18.2 years, suggesting prolonged periods of undetected transmission. Once established, clusters persisted for approximately 0.95 to 22.1 years, indicating sustained local transmission following the initial introductions (Fig 3B).

## Discussion

Among HIV-1 subtypes and CRFs, CRF01_AE remains predominant across Southeast and East Asia. In this study, we investigated the evolutionary dynamics driving the dissemination of CRF01_AE. Using available near-full-length genome (NFLG) sequences with documented sampling country and year, we reconstructed the evolutionary and geographic history of CRF01_AE and identified 10 well-supported phylogenetic clusters with inferred origins in Thailand, Vietnam, and China (Figs 2 and 3A; S1 Fig). Six clusters were confined to China in the available dataset (CRF01_1AE, CRF01_3AE–CRF01_4AE, CRF01_6AE–CRF01_8AE), two were identified in Thailand (CRF01_9AE and CRF01_10AE), one was shared between Vietnam and China (CRF01_2AE), and one was shared between Thailand and China (CRF01_5AE). The strong phylogenetic support and sustained circulation of these clusters indicate the establishment of distinct lineages implicated in extensive regional transmission, particularly within highly connected susceptible populations [42–45].

The emergence and subsequent spread of CRF01_AE in Southeast Asia have occurred against a background of increasing regional mobility and extensive sex-work networks. Economic constraints and limited industrialization have contributed to the development of tourism as an important sector in Thailand, alongside the growth of a large informal sex industry [46–48]. Transnational sex-work networks linking populations across Southeast Asia, Africa and other regions may have provided opportunities for virus introduction and subsequent dissemination. Within the present dataset, the most basal HIV-1 CRF01_AE sequences were sampled in the Central African Republic in 1990 (Fig 2; S1 Fig), consistent with previous evidence supporting an African origin for the CRF01_AE lineages that subsequently became established in Thailand [3,7,8].

The marked increase in CRF01_AE genetic diversity from 1984 to 1992 (S4 Fig) coincided with rapid epidemic expansion in Thailand and extensive heterosexual transmission involving sex-work networks [1,13,46,47,49,50]. These transmission networks may therefore have contributed to the early local amplification of CRF01_AE. The subsequent reduction in genetic diversity between 1992 and 1997 (S4 Fig) coincided with strengthened HIV prevention and control measures, including the 100% Condom Use Program [51–53], initiated in 1990 and expanded nationally in 1991, together with regulatory measures prohibiting prostitution in public places and brothels and imposing fines on individuals providing sexual services. A second increase in inferred genetic diversity between 1997 and 2006 (S4 Fig) occurred during a period of rapidly increasing outbound travel from China to Thailand and coincided with the inferred expansion and cross-border movement of multiple HIV-1 CRF01_AE lineages from Thailand to China [1,13]. Extensive sex-work networks in Thailand may also have provided opportunities for CRF01_AE introduction into Vietnam [17,54,55]. Concurrently, illicit-drug trafficking through the Golden Triangle, a major historical opium-producing region along the Thailand–Laos–Myanmar border, provided a regional context for the expansion of injecting-drug-use networks and associated HIV transmission in Vietnam [56]. Subsequent circulation through sexual and injecting-drug-use networks contributed to the emergence of CRF01_2AE in Vietnam, followed by the introduction of this lineage into China (Figs 2, 3A, and 5; S1 Fig, and S7 Fig) [1,13,57].

The sex industry in Thailand has contributed substantially to several pervasive public health challenges, including illicit drug use, sexually transmitted infections, and HIV transmission [58]. Continued expansion of this sector reflects complex economic, political, and social conditions and, in some settings, limited formal regulation [59]. Within this regional epidemiological context, CRF01_AE appears to have spread from Africa into Thailand and subsequently across Southeast Asia and China. The resulting descendant lineages have persisted for decades through a combination of introduction events and sustained local transmission, illustrating how early cross-border dissemination can generate long-lived regional transmission networks.

Collectively, our findings indicate that cross-border viral movement played a major role in the early dissemination of CRF01_AE across China and Southeast Asia, with Thailand and Vietnam representing key inferred sources of regional introductions (Figs 2–4). Interpretation of these findings is constrained by the restricted geographic and temporal representation of available CRF01_AE NFLG sequences. The present study focused on temporally informative sequences from Thailand and Vietnam in Southeast Asia and China in East Asia to characterize the early evolutionary history and geographic dissemination of CRF01_AE, but sequence availability remains limited for several other countries in the region. As such, broader and more systematic genomic surveillance, particularly across Southeast Asia, will be essential for refining inferences regarding geographic origins, migration histories, and transmission dynamics of CRF01_AE. Although some lineages appear to have become locally established, regional viral movement has contributed substantially to the broader epidemic landscape. Prolonged periods of cryptic transmission likely preceded cluster detection, consistent with limited early molecular surveillance and under-recognition of transmission within key populations (Fig 3). Once established, several transmission lineages persisted for decades, sustaining local epidemics and extending the epidemiological consequences of early introduction events. These findings emphasize the importance of timely molecular surveillance and coordinated regional monitoring for early detection of emerging lineages, disruption of transmission networks, and containment of cross-border HIV spread.

## Supporting information

S1 Fig Maximum-likelihood phylogeny of HIV-1 CRF01_AE. Phylogenetic tree inferred from near-complete genome sequences of HIV-1 CRF01_AE using a maximum-likelihood approach. The outermost circle indicates the sampling locations.
(PDF)

S2 Fig Average evolutionary divergence within and between HIV-1 CRF01_AE clusters. The matrix displays mean pairwise genetic distances, with intra-cluster divergence values shown along the diagonal and inter-cluster divergence values presented in the off-diagonal cells.
(PDF)

S3 Fig Temporal signal of HIV-1 CRF01_AE. Regression of root-to-tip genetic distances against sampling years for HIV-1 CRF01_AE. Colors denote distinct geographic locations, illustrating temporal structure in the dataset.
(PDF)

S4 Fig Bayesian Skygrid demographic reconstruction of HIV-1 CRF01_AE. The vertical axis represents the effective number of infections (Ne) multiplied by the mean viral generation time (τ). The solid line indicates the median, while the shaded region represents the 95% credibility interval of Neτ over time.
(PDF)

S5 Fig Temporal dynamics of trunk reward proportions for each geographic location. Colors represent distinct geographic locations. (PDF)

S6 Fig Cumulative Markov jumps from a given geographic location to another over time where each line represents a single phylogeny from the posterior sample of trees. Colors indicate distinct migration pathways between geographic locations.
(PDF)

S7 Fig Markov jump trajectory plot illustrating the inferred ancestral transitions between geographic locations, from the Central African Republic to the sampling location of isolate 05GX001 of HIV-1 CRF01_2AE. The trajectories are derived from the posterior distribution of phylogenetic trees annotated with Markov jump histories. Horizontal lines represent time intervals during which a specific geographic location is maintained in the spatiotemporal ancestry of the virus, whereas vertical lines indicate discrete geographic location transitions (Markov jumps). The density of the lines reflects the posterior uncertainty associated with the inferred locations and transition timings.
(PDF)

## References

[1] Li X, Liu H, Liu L, Feng Y, Kalish ML, Ho SYW, et al. Tracing the epidemic history of HIV-1 CRF01_AE clusters using near-complete genome sequences. Sci Rep. 2017;7(1):4024. doi: 10.1038/s41598-017-03820-8 28642469 PMC5481428

[2] Vrancken B, Zhao B, Li X, Han X, Liu H, Zhao J, et al. Comparative circulation dynamics of the five main HIV types in China. J Virol. 2020;94(23):e00683-20. doi: 10.1128/JVI.00683-20 32938762 PMC7654276

[3] Gao F, Robertson DL, Morrison SG, Hui H, Craig S, Decker J, et al. The heterosexual human immunodeficiency virus type 1 epidemic in Thailand is caused by an intersubtype (A/E) recombinant of African origin. J Virol. 1996;70(10):7013–29. doi: 10.1128/JVI.70.10.7013-7029.1996 8794346 PMC190752

[4] Anderson JP, Rodrigo AG, Learn GH, Madan A, Delahunty C, Coon M, et al. Testing the hypothesis of a recombinant origin of human immunodeficiency virus type 1 subtype E. J Virol. 2000;74(22):10752–65. doi: 10.1128/jvi.74.22.10752-10765.2000 11044120 PMC110950

[5] Robertson DL, Anderson JP, Bradac JA, Carr JK, Foley B, Funkhouser RK, et al. HIV-1 nomenclature proposal. Science. 2000;288(5463):55–6. doi: 10.1126/science.288.5463.55d 10766634

[6] Kalish ML, Robbins KE, Pieniazek D, Schaefer A, Nzilambi N, Quinn TC. Recombinant viruses and early global HIV-1 epidemic. Emerg Infect Dis. 2004;10:1227–34. doi: 10.3201/eid1007.030904 PMC3323344 15324542

[7] McCutchan FE, Hegerich PA, Brennan TP, Phanuphak P, Singharaj P, Jugsudee A, et al. Genetic variants of HIV-1 in Thailand. AIDS Res Hum Retroviruses. 1992;8(11):1887–95. doi: 10.1089/aid.1992.8.1887 1489577

[8] Carr JK, Salminen MO, Koch C, Gotte D, Artenstein AW, Hegerich PA, et al. Full-length sequence and mosaic structure of a human immunodeficiency virus type 1 isolate from Thailand. J Virol. 1996;70(9):5935–43. doi: 10.1128/JVI.70.9.5935-5943.1996 8709215 PMC190613

[9] Nelson KE, Celentano DD, Suprasert S, Wright N, Eiumtrakul S, Tulvatana S, et al. Risk factors for HIV infection among young adult men in northern Thailand. JAMA. 1993;270(8):955–60. doi: 10.1001/jama.1993.03510080059032 8345647

[10] Ou CY, Takebe Y, Luo CC, Kalish M, Auwanit W, Bandea C, et al. Wide distribution of two subtypes of HIV-1 in Thailand. AIDS Res Hum Retroviruses. 1992;8(8):1471–2. doi: 10.1089/aid.1992.8.1471 1466984

[11] Kilmarx PH, Supawitkul S, Wankrairoj M, Uthaivoravit W, Limpakarnjanarat K, Saisorn S, et al. Explosive spread and effective control of human immunodeficiency virus in northernmost Thailand: The epidemic in Chiang Rai province, 1988-99. AIDS. 2000;14: 2731–40. doi: 10.1097/00002030-200012010-00013 11125892

[12] Liao H, Tee KK, Hase S, Uenishi R, Li X-J, Kusagawa S, et al. Phylodynamic analysis of the dissemination of HIV-1 CRF01_AE in Vietnam. Virology. 2009;391(1):51–6. doi: 10.1016/j.virol.2009.05.023 19540543

[13] Feng Y, He X, Hsi JH, Li F, Li X, Wang Q, et al. The rapidly expanding CRF01_AE epidemic in China is driven by multiple lineages of HIV-1 viruses introduced in the 1990s. AIDS. 2013;27(11):1793–802. doi: 10.1097/QAD.0b013e328360db2d 23807275 PMC3819312

[14] McCutchan FE, Artenstein AW, Sanders-Buell E, Salminen MO, Carr JK, Mascola JR, et al. Diversity of the envelope glycoprotein among human immunodeficiency virus type 1 isolates of clade E from Asia and Africa. J Virol. 1996;70(6):3331–8. doi: 10.1128/JVI.70.6.3331-3338.1996 8648662 PMC190203

[15] Chen JHK, Wong KH, Li P, Chan KC, Lee MP, Lam HY, et al. Molecular epidemiological study of HIV-1 CRF01_AE transmission in Hong Kong. J Acquir Immune Defic Syndr. 2009;51(5):530–5. doi: 10.1097/QAI.0b013e3181aac516 19521252

[16] Abubakar YF, Meng Z, Zhang X, Xu J. Multiple independent introductions of HIV-1 CRF01_AE identified in China: what are the implications for prevention?. PLoS One. 2013;8(11):e80487. doi: 10.1371/journal.pone.0080487 24282546 PMC3839914

[17] Angelis K, Albert J, Mamais I, Magiorkinis G, Hatzakis A, Hamouda O, et al. Global dispersal pattern of HIV Type 1 subtype CRF01_AE: A genetic trace of human mobility related to heterosexual sexual activities centralized in Southeast Asia. J Infect Dis. 2015;211(11):1735–44. doi: 10.1093/infdis/jiu666 25512631

[18] An M, Han X, Xu J, Chu Z, Jia M, Wu H, et al. Reconstituting the epidemic history of HIV strain CRF01_AE among men who have sex with men (MSM) in Liaoning, northeastern China: Implications for the expanding epidemic among MSM in China. J Virol. 2012;86(22):12402–6. doi: 10.1128/JVI.00262-12 22933290 PMC3486469

[19] Ye J, Xin R, Yu S, Bai L, Wang W, Wu T, et al. Phylogenetic and temporal dynamics of human immunodeficiency virus type 1 CRF01_AE in China. PLoS One. 2013;8(1):e54238. doi: 10.1371/journal.pone.0054238 23365653 PMC3554705

[20] Argimón S, Abudahab K, Goater RJE, Fedosejev A, Bhai J, Glasner C, et al. Microreact: Visualizing and sharing data for genomic epidemiology and phylogeography. Microb Genom. 2016;2(11):e000093. doi: 10.1099/mgen.0.000093 28348833 PMC5320705

[21] Guindon S, Dufayard J-F, Lefort V, Anisimova M, Hordijk W, Gascuel O. New algorithms and methods to estimate maximum-likelihood phylogenies: Assessing the performance of PhyML 3.0. Syst Biol. 2010;59(3):307–21. doi: 10.1093/sysbio/syq010 20525638

[22] Ragonnet-Cronin M, Hodcroft E, Hué S, Fearnhill E, Delpech V, Brown AJL. Automated analysis of phylogenetic clusters. BMC Bioinform. 2013;14:317. doi: 10.1186/1471-2105-14-317 PMC4228337 24191891

[23] Kumar S, Stecher G, Suleski M, Sanderford M, Sharma S, Tamura K. MEGA12: Molecular evolutionary genetic analysis version 12 for adaptive and green computing. Mol Biol Evol. 2024;41(12):msae263. doi: 10.1093/molbev/msae263 39708372 PMC11683415

[24] Stecher G, Tamura K, Kumar S. Mol Biol Evol. 2020;37:1237–9. doi: 10.1093/molbev/msz312 PMC7086165 31904846

[25] Tamura K, Nei M, Kumar S. Prospects for inferring very large phylogenies by using the neighbor-joining method. Proc Natl Acad Sci U S A. 2004;101(30):11030–5. doi: 10.1073/pnas.0404206101 15258291 PMC491989

[26] Tamura K, Kumar S. Evolutionary distance estimation under heterogeneous substitution pattern among lineages. Mol Biol Evol. 2002;19:1727–36. doi: 10.1093/oxfordjournals.molbev.a003995 12270899

[27] Rambaut A, Lam TT, Max Carvalho L, Pybus OG. Exploring the temporal structure of heterochronous sequences using TempEst (formerly Path-O-Gen). Virus Evol. 2016;2(1):vew007. doi: 10.1093/ve/vew007 27774300 PMC4989882

[28] Drummond AJ, Ho SYW, Phillips MJ, Rambaut A. Relaxed phylogenetics and dating with confidence. PLoS Biol. 2006;4(5):e88. doi: 10.1371/journal.pbio.0040088 16683862 PMC1395354

[29] Gill MS, Lemey P, Faria NR, Rambaut A, Shapiro B, Suchard MA. Improving Bayesian population dynamics inference: A coalescent-based model for multiple loci. Mol Biol Evol. 2013;30(3):713–24. doi: 10.1093/molbev/mss265 23180580 PMC3563973

[30] Suchard MA, Lemey P, Baele G, Ayres DL, Drummond AJ, Rambaut A. Bayesian phylogenetic and phylodynamic data integration using BEAST 1.10. Virus Evolution. 2018;4. doi: 10.1093/ve/vey016 PMC6007674 29942656

[31] Rambaut A, Drummond AJ, Xie D, Baele G, Suchard MA. Posterior summarization in bayesian phylogenetics using tracer 1.7. Syst Biol. 2018;67(5):901–4. doi: 10.1093/sysbio/syy032 29718447 PMC6101584

[32] Lemey P, Rambaut A, Drummond AJ, Suchard MA. Bayesian phylogeography finds its roots. PLoS Comput Biol. 2009;5(9):e1000520. doi: 10.1371/journal.pcbi.1000520 19779555 PMC2740835

[33] Bielejec F, Baele G, Vrancken B, Suchard MA, Rambaut A, Lemey P. SpreaD3: Interactive visualization of spatiotemporal history and trait evolutionary processes. Mol Biol Evol. 2016;33(8):2167–9. doi: 10.1093/molbev/msw082 27189542 PMC6398721

[34] Kass RE, Raftery AE. Bayes factors. J Am Stat Assoc. 1995;90(430):773–95. doi: 10.1080/01621459.1995.10476572

[35] Minin VN, Suchard MA. Fast, accurate and simulation-free stochastic mapping. Philos Trans R Soc Lond B Biol Sci. 2008;363(1512):3985–95. doi: 10.1098/rstb.2008.0176 18852111 PMC2607419

[36] O’Brien JD, Minin VN, Suchard MA. Learning to count: Robust estimates for labeled distances between molecular sequences. Mol Biol Evol. 2009;26(4):801–14. doi: 10.1093/molbev/msp003 19131426 PMC2734148

[37] Minin VN, Suchard MA. Counting labeled transitions in continuous-time Markov models of evolution. J Math Biol. 2008;56(3):391–412. doi: 10.1007/s00285-007-0120-8 17874105

[38] Lemey P, Ruktanonchai N, Hong SL, Colizza V, Poletto C, Van den Broeck F, et al. Untangling introductions and persistence in COVID-19 resurgence in Europe. Nature. 2021;595(7869):713–7. doi: 10.1038/s41586-021-03754-2 34192736 PMC8324533

[39] Yu G, Lam TT-Y, Zhu H, Guan Y. Two methods for mapping and visualizing associated data on phylogeny using *Ggtree*. Mol Biol Evol. 2018;35(12):3041–3. doi: 10.1093/molbev/msy194 30351396 PMC6278858

[40] Yu G. Using ggtree to visualize data on tree-like structures. Curr Protoc Bioinformatics. 2020;69(1):e96. doi: 10.1002/cpbi.96 32162851

[41] Xu S, Li L, Luo X, Chen M, Tang W, Zhan L, et al. Ggtree: A serialized data object for visualization of a phylogenetic tree and annotation data. Imeta. 2022;1(4):e56. doi: 10.1002/imt2.56 38867905 PMC10989815

[42] Bengtsson L, Lu X, Liljeros F, Thanh HH, Thorson A. Strong propensity for HIV transmission among men who have sex with men in Vietnam: Behavioural data and sexual network modelling. BMJ Open. 2014;4(1):e003526. doi: 10.1136/bmjopen-2013-003526 24435887 PMC3902196

[43] Young AM, Jonas AB, Mullins UL, Halgin DS, Havens JR. Network structure and the risk for HIV transmission among rural drug users. AIDS Behav. 2013;17(7):2341–51. doi: 10.1007/s10461-012-0371-2 23184464 PMC3600060

[44] Rothenberg RB, Potterat JJ, Woodhouse DE, Muth SQ, Darrow WW, Klovdahl AS. Social network dynamics and HIV transmission. AIDS. 1998;12(12):1529–36. doi: 10.1097/00002030-199812000-00016 9727575

[45] De P, Singh AE, Wong T, Yacoub W, Jolly AM. Sexual network analysis of a gonorrhoea outbreak. Sex Transm Infect. 2004;80(4):280–5. doi: 10.1136/sti.2003.007187 15295126 PMC1744860

[46] Bamber SD, Hewison KJ, Underwood PJ. A history of sexually transmitted diseases in Thailand: Policy and politics. Genitourin Med. 1993;69(2):148–57. doi: 10.1136/sti.69.2.148 8509097 PMC1195049

[47] Leheny D. A political economy of Asian sex tourism. Annal Tourism Res. 1995;22(2):367–84. doi: 10.1016/0160-7383(94)00082-4

[48] Van Kerkwijk C. Sex tourism in Thailand. AIDS Soc. 1992;4(1):6–7. 12286018

[49] Koetsawang S. AIDS in Asia. Trop Med Parasitol. 1993;44(2):127–8. 8367661

[50] Hung J. Pragmatic approaches to control inbound sex tourism and prostitution in Thailand and Cambodia: the examination of relevant policy gaps and suggestion of multifaceted policy development. Int J Soc Sci Policy. 2023;43:1175–89. doi: 10.1108/IJSSP-03-2023-0048

[51] Rojanapithayakorn W, Hanenberg R. The 100% condom program in Thailand. AIDS. 1996;10(1):1–7. doi: 10.1097/00002030-199601000-00001 8924236

[52] Celentano DD, Nelson KE, Suprasert S, Eiumtrakul S, Tulvatana S, Kuntolbutra S, et al. Risk factors for HIV-1 seroconversion among young men in Northern Thailand. JAMA. 1996;275(2):122–7. doi: 10.1001/jama.1996.03530260036028 8531307

[53] Hanenberg RS, Rojanapithayakorn W, Kunasol P, Sokal DC. Impact of Thailand’s HIV-control programme as indicated by the decline of sexually transmitted diseases. Lancet. 1994;344(8917):243–5. doi: 10.1016/s0140-6736(94)93004-x 7913163

[54] Menu E, Truong TX, Lafon ME, Nguyen TH, Müller-Trutwin MC, Nguyen TT, et al. HIV type 1 Thai subtype E is predominant in South Vietnam. AIDS Res Hum Retroviruses. 1996;12(7):629–33. doi: 10.1089/aid.1996.12.629 8743088

[55] Khairunisa SQ, Indriati DW, Megasari NLA, Ueda S, Kotaki T, Fahmi M, et al. Spatial-temporal transmission dynamics of HIV-1 CRF01_AE in Indonesia. Sci Rep. 2024;14(1):9917. doi: 10.1038/s41598-024-59820-y 38730038 PMC11087524

[56] Beyrer C. Human immunodeficiency virus (HIV) infection rates and heroin trafficking: Fearful symmetries. In: Thomas YF, Richardson D, Cheung I, editors. Geography and drug addiction. Dordrecht: Springer Netherlands; 2008. 243–54. doi: 10.1007/978-1-4020-8509-3_15

[57] Zhang Y, Lu L, Ba L, Liu L, Yang L, Jia M, et al. Dominance of HIV-1 subtype CRF01_AE in sexually acquired cases leads to a new epidemic in Yunnan province of China. PLoS Med. 2006;3(11):e443. doi: 10.1371/journal.pmed.0030443 17105339 PMC1635743

[58] Ford N, Koetsawang S. The socio-cultural context of the transmission of HIV in Thailand. Soc Sci Med. 1991;33(4):405–14. doi: 10.1016/0277-9536(91)90321-3 1948153

[59] Huda S. Sex trafficking in South Asia. Int J Gynaecol Obstet. 2006;94(3):374–81. doi: 10.1016/j.ijgo.2006.04.027 16846602

