# Supplementary material for: Global spread and evolutionary trajectories of HIV-1 CRF01_AE: Insights from phylogenetic analysis
Source: PLoS One. 2026 Oct 8;21(10):e0360404. doi: 10.1371/journal.pone.0360404 (PMC13649028; doi:10.1371/journal.pone.0360404)
Supplement: Supplementary file 1 [file pone.0360404.s001.pdf]

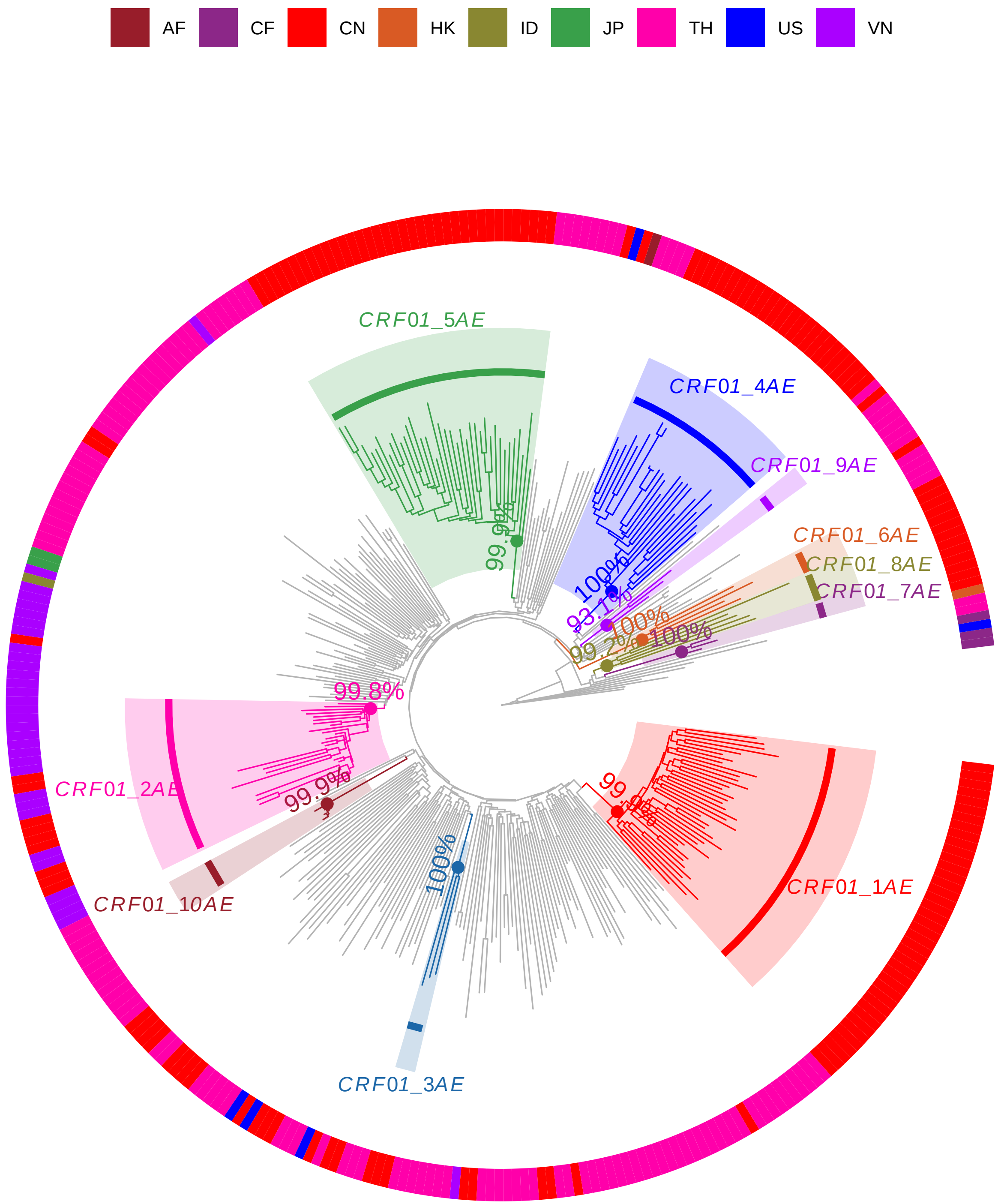
AF
CF
CN
HK
ID
JP
TH
US
VN
CRF01_5AE
CRF01_4AE
CRF01_9AE
CRF01_6AE
CRF01_8AE
CRF01_7AE
CRF01_2AE
CRF01_10AE
CRF01_3AE
CRF01_1AE
99.8%
99.9%
100%
93.1%
99.2%
100%
100%
100%
