# Supplementary material for: Global spread and evolutionary trajectories of HIV-1 CRF01_AE: Insights from phylogenetic analysis
Source: PLoS One. 2026 Oct 8;21(10):e0360404. doi: 10.1371/journal.pone.0360404 (PMC13649028; doi:10.1371/journal.pone.0360404)
Supplement: Supplementary file 2 [file pone.0360404.s002.pdf]

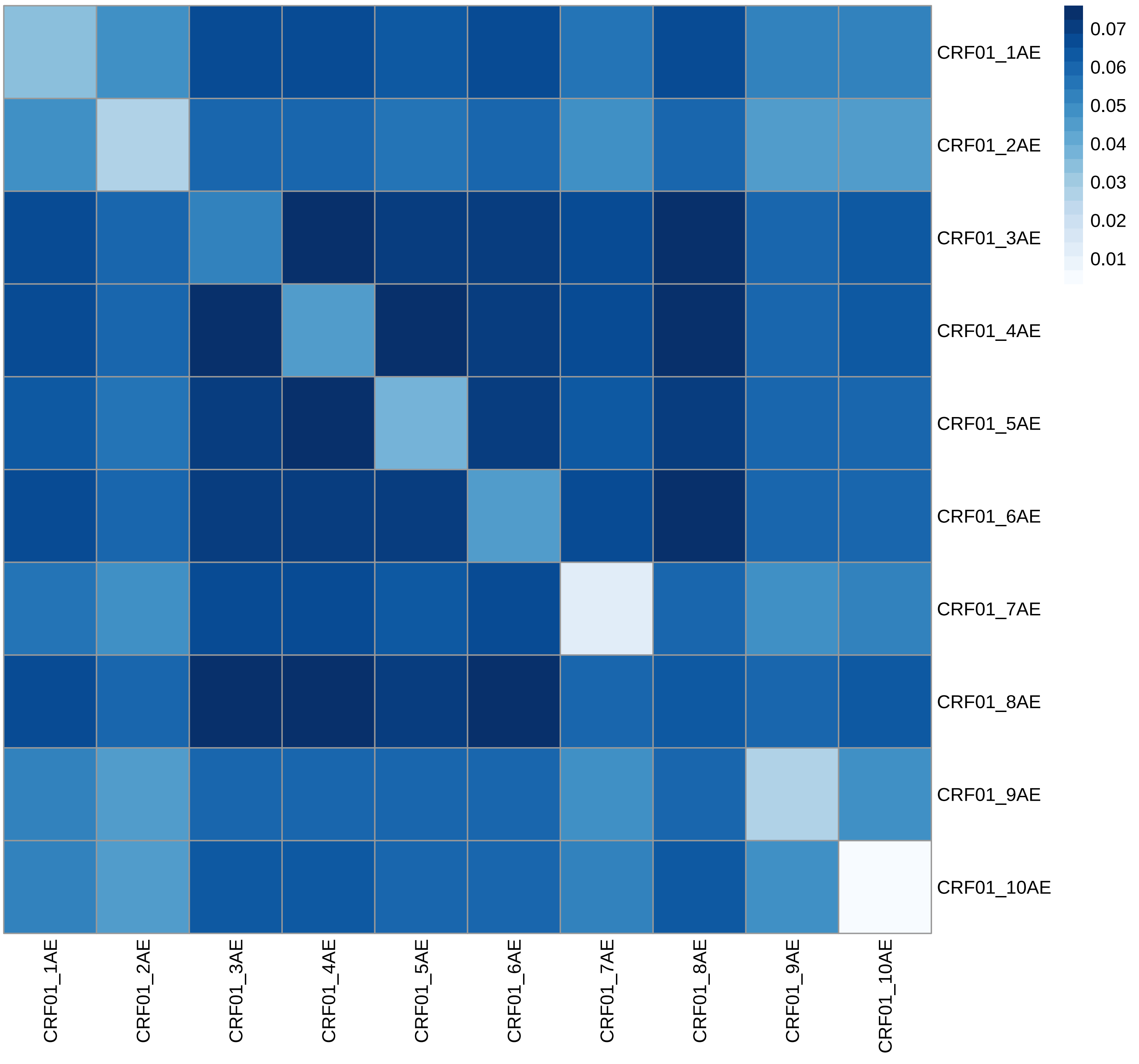
0.07
0.06
0.05
0.04
0.03
0.02
0.01
CRF01_1AE
CRF01_2AE
CRF01_3AE
CRF01_4AE
CRF01_5AE
CRF01_6AE
CRF01_7AE
CRF01_8AE
CRF01_9AE
CRF01_10AE
