# Supplementary material for: Global spread and evolutionary trajectories of HIV-1 CRF01_AE: Insights from phylogenetic analysis
Source: PLoS One. 2026 Oct 8;21(10):e0360404. doi: 10.1371/journal.pone.0360404 (PMC13649028; doi:10.1371/journal.pone.0360404)
Supplement: Supplementary file 3 [file pone.0360404.s003.pdf]

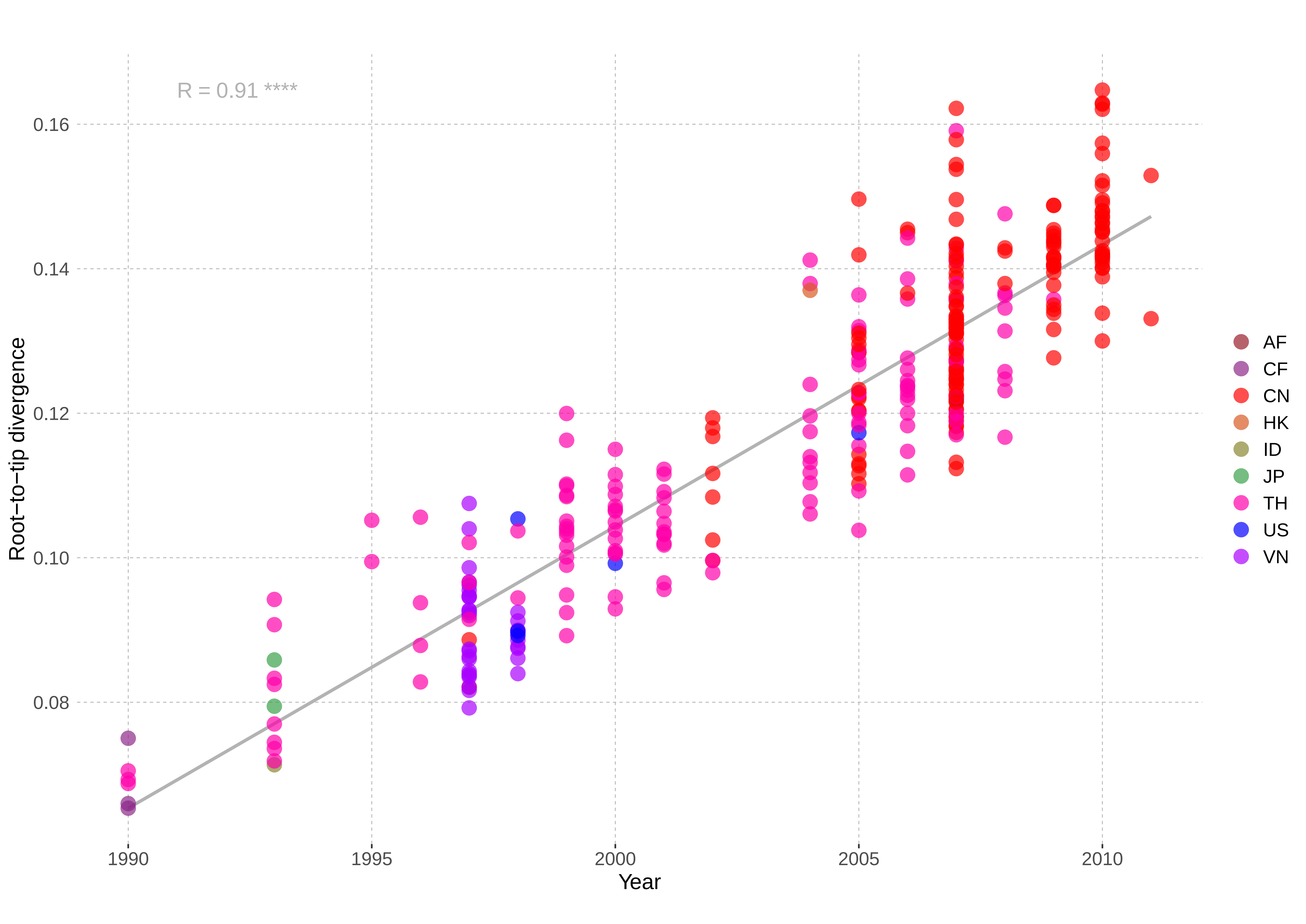

R = 0.91 ****
Root−to−tip divergence
0.16
0.14
0.12
0.10
0.08
1990
1995
2000
2005
2010
Year
AF
CF
CN
HK
ID
JP
TH
US
VN
