# Supplementary material for: Global spread and evolutionary trajectories of HIV-1 CRF01_AE: Insights from phylogenetic analysis
Source: PLoS One. 2026 Oct 8;21(10):e0360404. doi: 10.1371/journal.pone.0360404 (PMC13649028; doi:10.1371/journal.pone.0360404)
Supplement: Supplementary file 4 [file pone.0360404.s004.pdf]

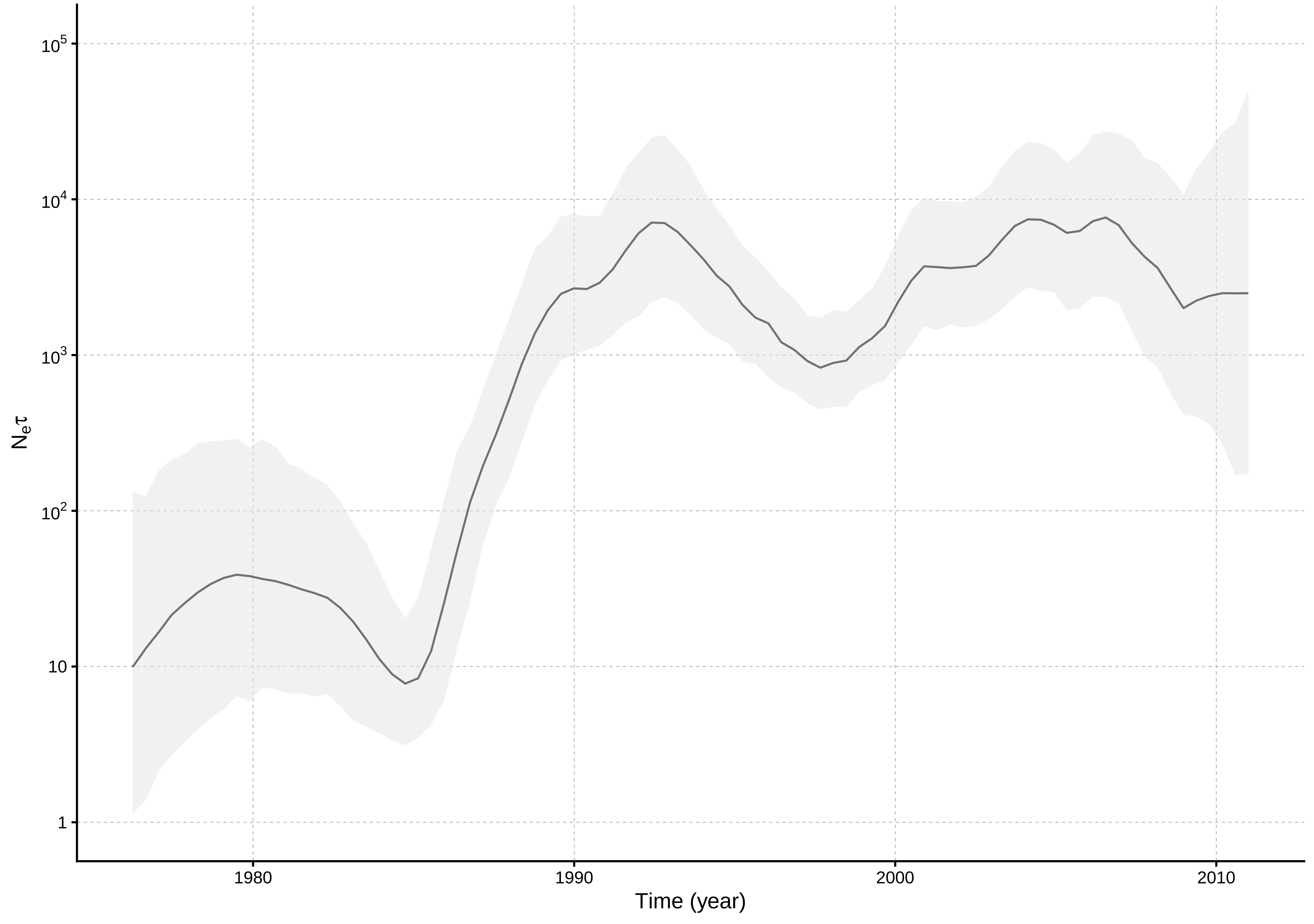

10^5
10^4
10^3
10^2
10
1
$N_e\tau$
1980
1990
2000
2010
Time (year)
