# Supplementary material for: Global spread and evolutionary trajectories of HIV-1 CRF01_AE: Insights from phylogenetic analysis
Source: PLoS One. 2026 Oct 8;21(10):e0360404. doi: 10.1371/journal.pone.0360404 (PMC13649028; doi:10.1371/journal.pone.0360404)
Supplement: Supplementary file 5 [file pone.0360404.s005.pdf]

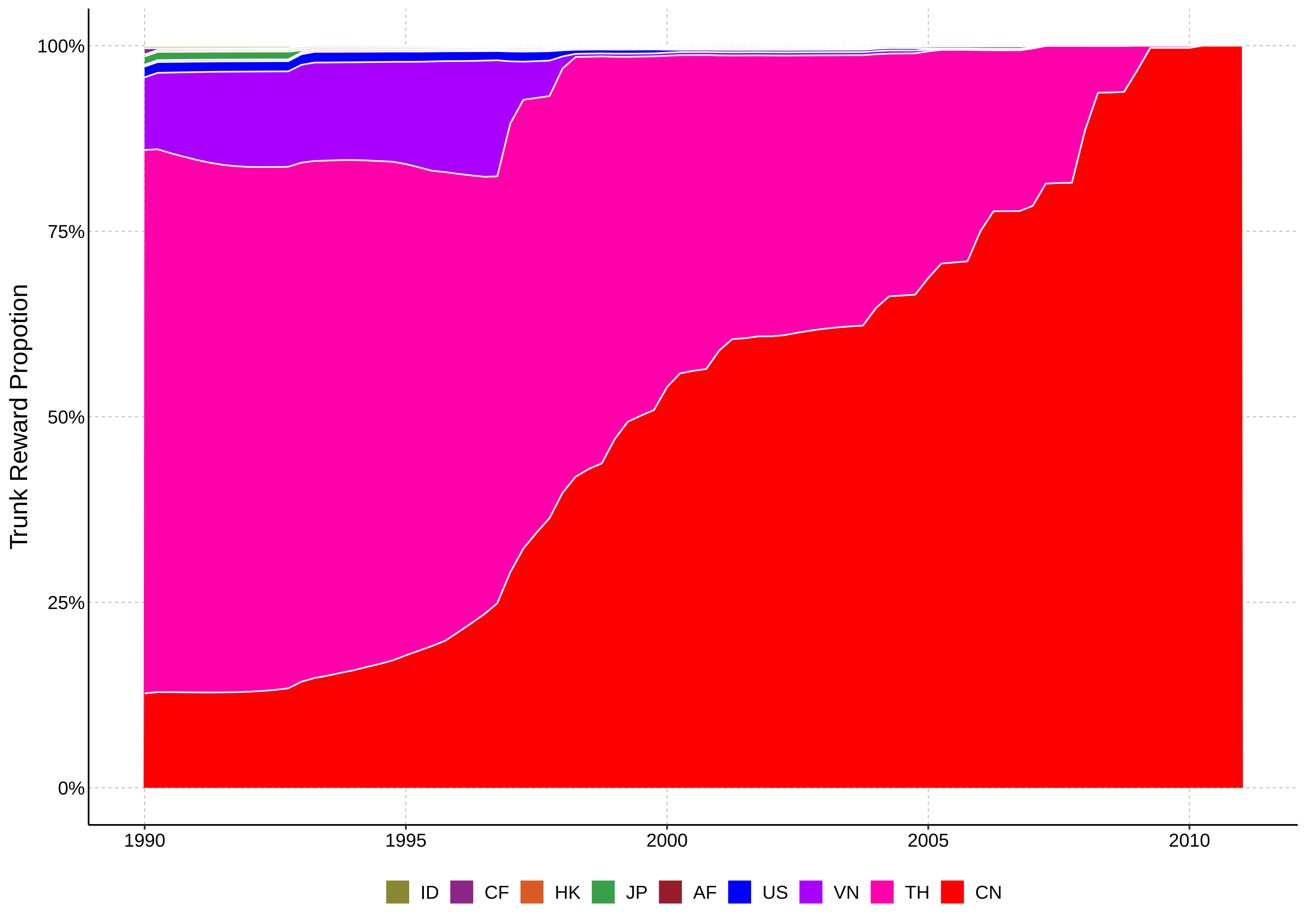

Trunk Reward Propotion
100%
75%
50%
25%
0%
1990
1995
2000
2005
2010
ID
CF
HK
JP
AF
US
VN
TH
CN
