# Supplementary material for: Global spread and evolutionary trajectories of HIV-1 CRF01_AE: Insights from phylogenetic analysis
Source: PLoS One. 2026 Oct 8;21(10):e0360404. doi: 10.1371/journal.pone.0360404 (PMC13649028; doi:10.1371/journal.pone.0360404)
Supplement: Supplementary file 7 [file pone.0360404.s007.pdf]

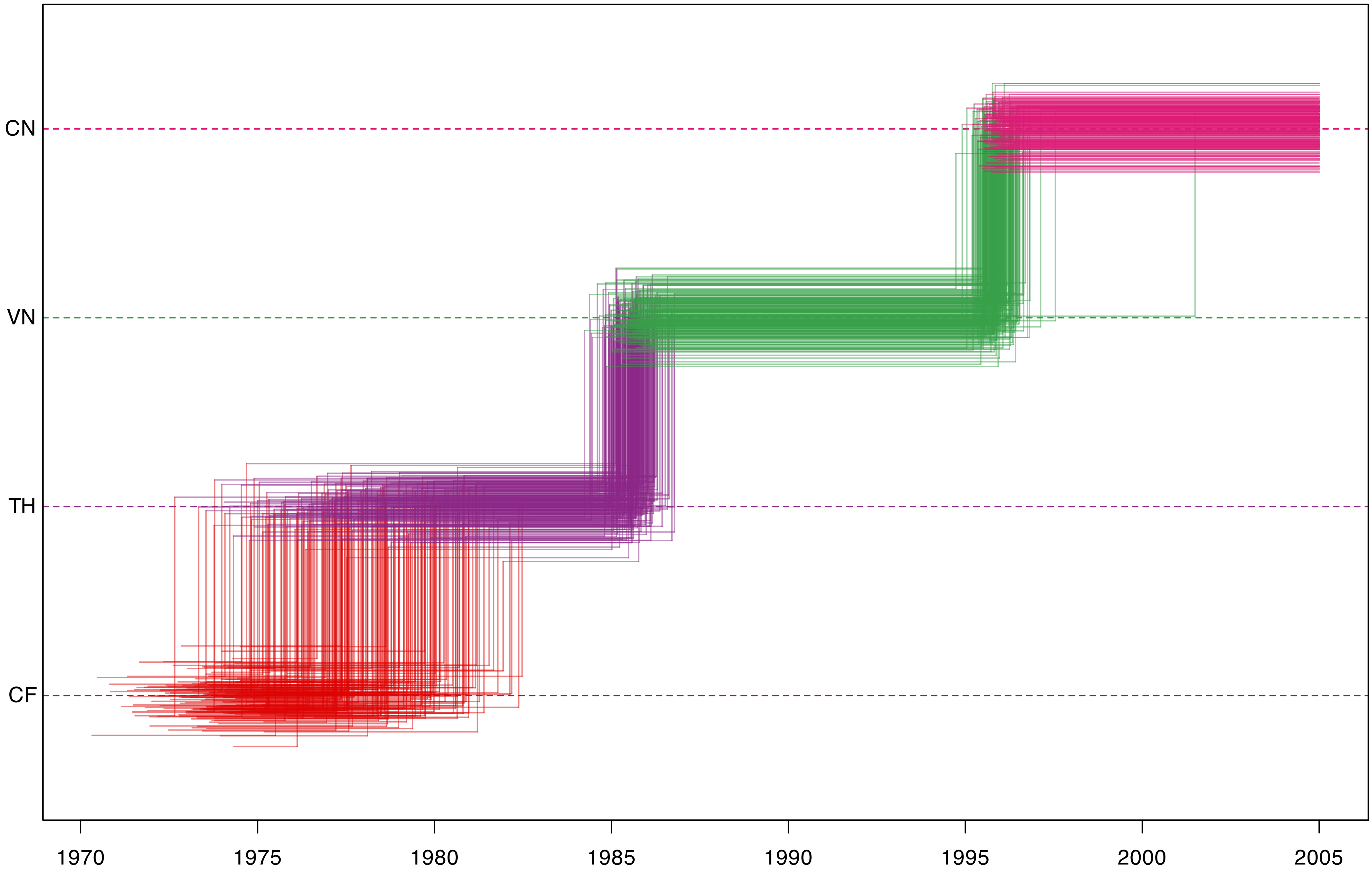
CN
VN
TH
CF
1970
1975
1980
1985
1990
1995
2000
2005
